# Remimazolam for simultaneous percutaneous mitral valve clip and percutaneous left atrial appendage closure in an elderly patient with impaired cardiac function: A case report

**DOI:** 10.1002/ccr3.9215

**Published:** 2024-07-21

**Authors:** Sumika Yamaguchi, Yusuke Ishida, Tomomi Sasaki, Satoshi Higuchi, Kiyoko Bito, Katsunori Oe

**Affiliations:** ^1^ Department of Anesthesiology Showa University School of Medicine Tokyo Japan; ^2^ Department of Anesthesiology Tokyo Saiseikai Central Hospital Tokyo Japan

**Keywords:** mitral regurgitation, patient state index, percutaneous left atrial appendage closure, remimazolam, transcatheter mitral valve repair

## Abstract

Remimazolam is a short‐acting benzodiazepine sedative with a short half‐life and little circulatory depression. The safe use of remimazolam in the anesthetic management of an elderly patient with impaired cardiac function is reported. The patient's hemodynamics remained stable, and the patient was managed without serious complications. Remimazolam may be an option for sedation in elderly patients with reduced cardiac function.

## 1 INTRODUCTION

In recent years, advances in transcatheter treatments have made it possible to perform them even in patients who cannot tolerate open heart surgery for valve replacement or valvuloplasty. Percutaneous mitral valve clip (MVC) therapy is one such transcatheter treatment for severe mitral regurgitation (MR).^1^ Percutaneous MVC therapy is often indicated for elderly patients, those with impaired cardiac function, and those who are not candidates for open heart surgery. Although it is minimally invasive, there is a risk of hemodynamic instability, since patients may lack compensatory mechanisms, and anesthesia may easily lead to circulatory collapse. However, in 2020, the use of remimazolam, a sedative with minimal hemodynamic effects, was approved in Japan,^2^ and there have been increasing reports of its use in various clinical scenarios. The anesthesia management of percutaneous MVC and percutaneous left atrial appendage closure (LAAC) in an elderly patient with impaired cardiac function is reported. Written, informed consent was obtained from the patient to publish this case report. The authors have no conflicts of interest to disclose.

## 2 CASE HISTORY/EXAMINATION

The patient was a 91‐year‐old woman with a height of 131.0 cm and weight of 34.5 kg. She had a history of cerebral infarction 8 years ago. She had been experiencing chest discomfort and dyspnea for approximately 1 year, with symptoms gradually worsening over time. She was initially seen by a local physician and subsequently transferred to our hospital with a diagnosis of paroxysmal atrial fibrillation. She was admitted with a diagnosis of exacerbation of heart failure secondary to chronic paroxysmal atrial fibrillation. During hospitalization, sinus pauses and sinus bradycardia were observed, leading to the insertion of a pacemaker. A transthoracic echocardiogram performed during hospitalization showed left atrial enlargement and moderate‐severe MR. However, heart failure symptoms improved with medical therapy, and she was discharged for outpatient follow‐up. After discharge, the patient was prescribed diuretics and remained asymptomatic regarding heart failure symptoms, but over time, there was an increase in heart size and brain natriuretic peptide levels, and stress echocardiography showed worsening of MR. Mitral valve repair was considered. In addition, she developed a cerebral hemorrhage during anticoagulant therapy 2 months before surgery was scheduled leading to a high risk for further hemorrhage. Therefore, LAAC was also considered. Considering the patient's age and overall condition, a decision was made to perform simultaneous percutaneous MVC and percutaneous LAAC as minimally invasive treatments.

**FIGURE 1:**
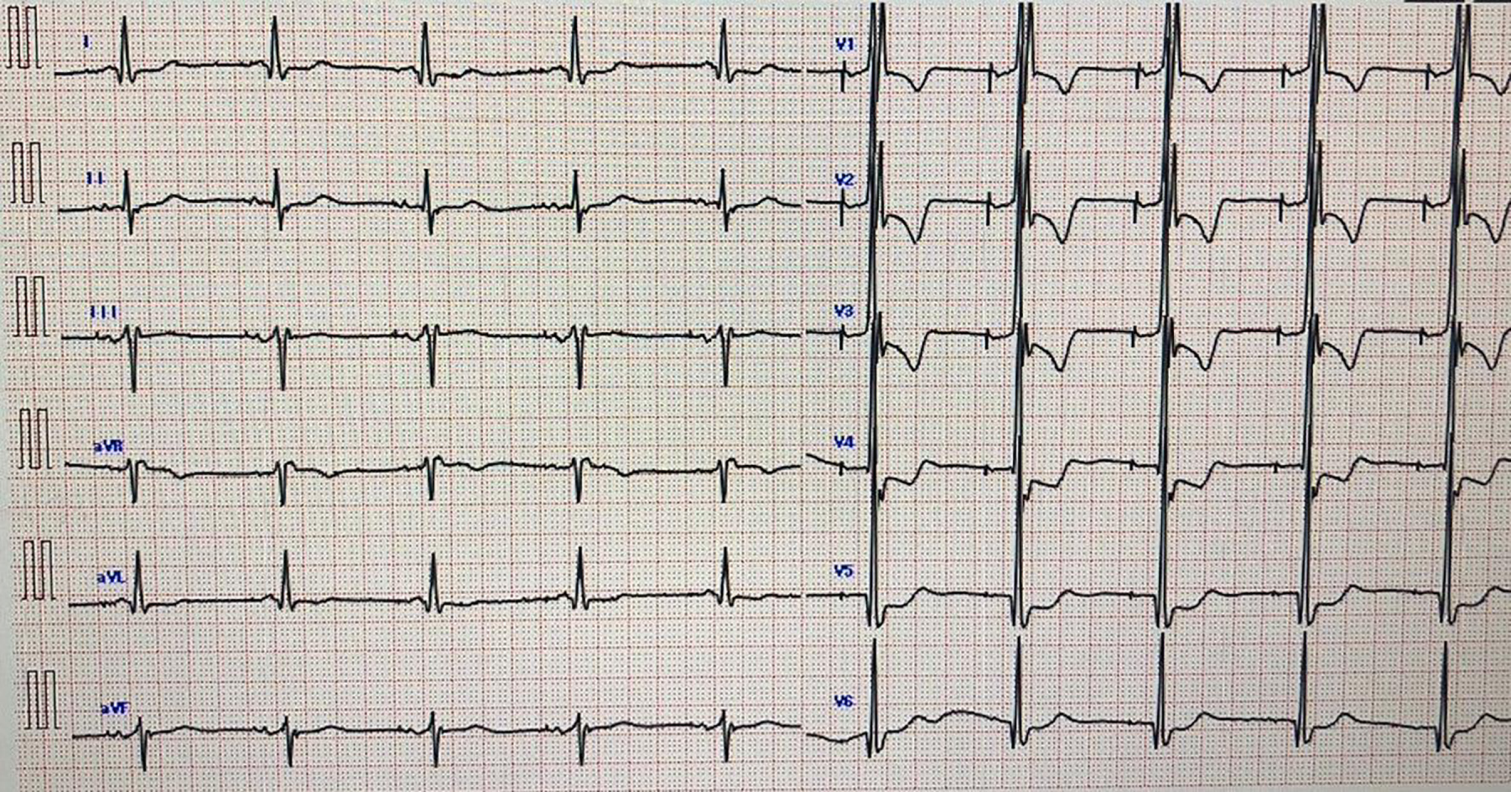
Electrocardiogram. Heart rate was 60 beats/min.

**FIGURE 2:**
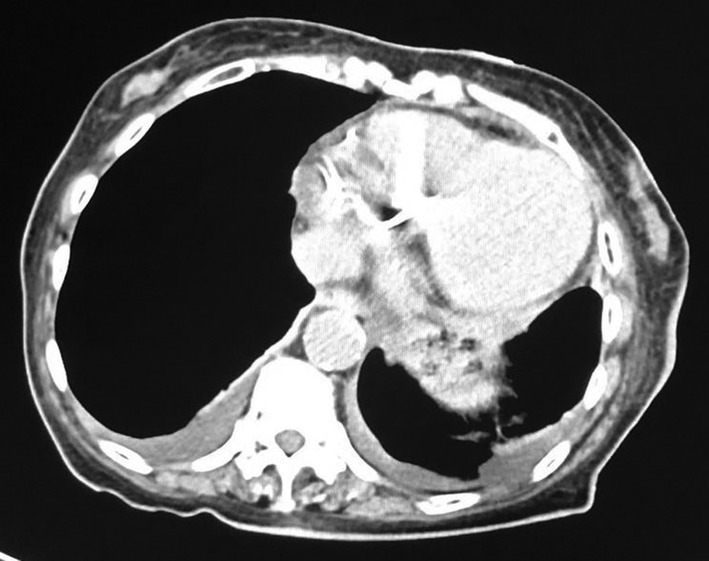
Computed tomography. Preoperatively, there was a small amount of pleural effusion.

**FIGURE 3:**
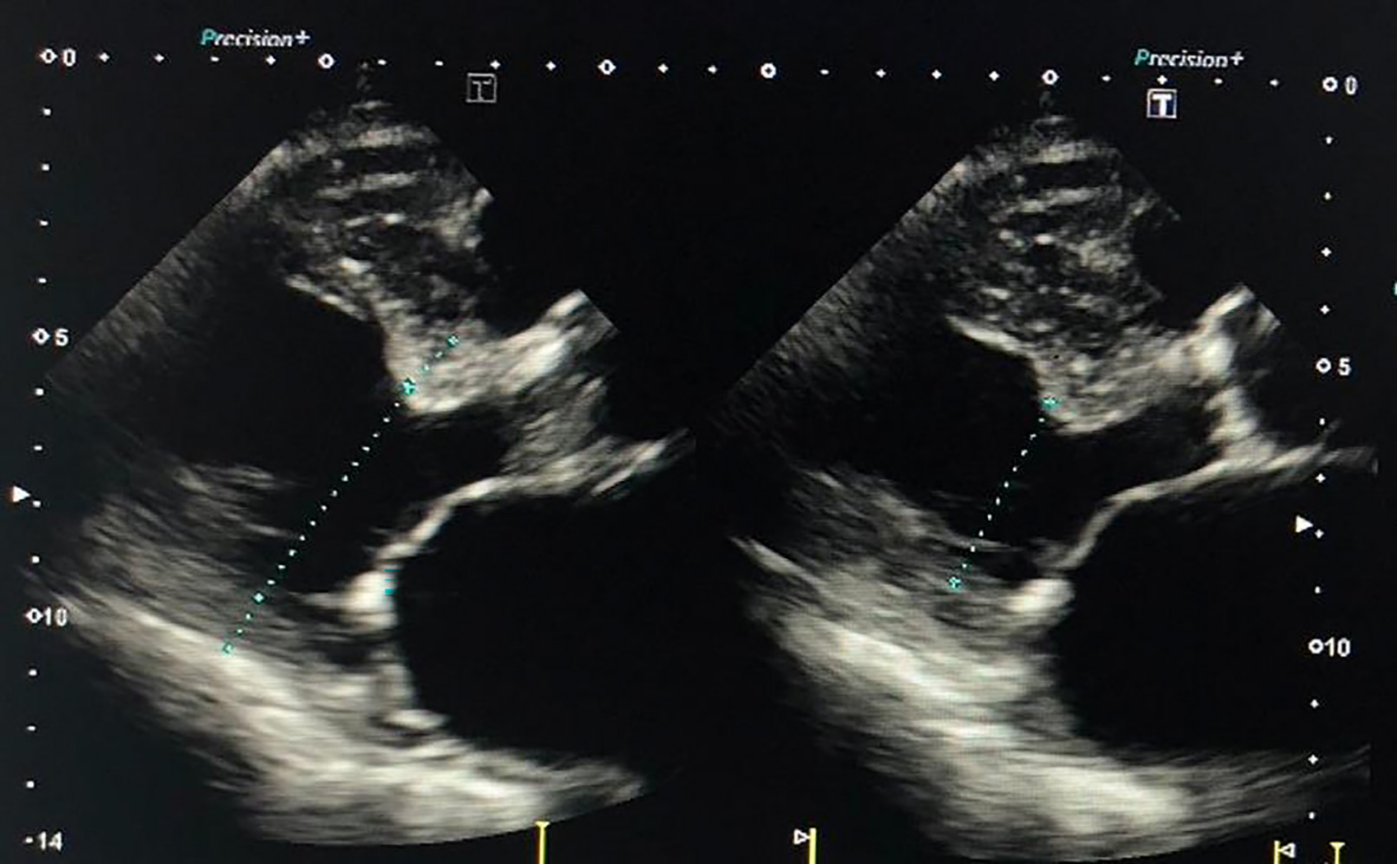
Transthoracic echocardiography. On transthoracic echocardiography, left ventricular end‐diastolic dimension/end‐systolic dimension was 46/36 mm. Diffuse moderate systolic dysfunction of ventricular wall motion was noted.

The patient was classified as New York Heart Association functional class II, with a blood pressure of 123/71 mmHg, heart rate of 60 bpm, and SpO_2_ of 97% (room air) (Figure 1). Thoracic computed tomography showed a small amount of pleural effusion (Figure 2). On transthoracic echocardiography, left ventricular ejection fraction was 39%, with left ventricular end‐diastolic dimension/end‐systolic dimension of 46/36 mm (Figure 3). Diffuse moderate systolic dysfunction of ventricular wall motion was noted. Tricuspid regurgitation was mild, whereas MR was severe. There was no evidence of compression across the ventricular septum.

## 3 METHODS (DIFFERENTIAL DIAGNOSIS, INVESTIGATIONS AND TREATMENT)

**FIGURE 4:**
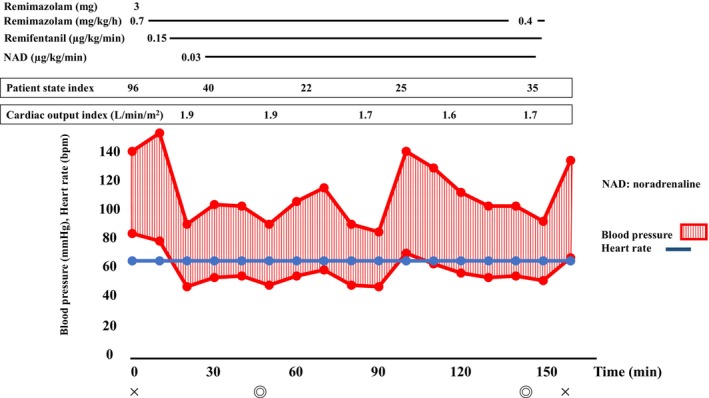
Patient's anesthesia record. Cardiovascular dynamics were stable in the management of anesthesia with remimazolam.

**FIGURE 5:**
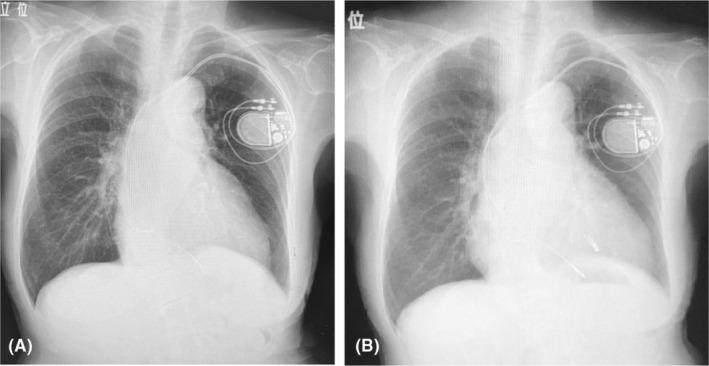
Chest x‐ray. Preoperatively, the cardiothoracic ratio is approximately 59%, indicating mild cardiac enlargement. In addition, a pacemaker has been inserted (A). There was no change at the time of discharge (B).

Anesthesia was induced with 3 mg (0.1 mg/kg) of remimazolam, 0.15 μg/kg/min of remifentanil, and 50 mg of rocuronium. Maintenance was achieved with 0.7 mg/kg/h of remimazolam, 0.15 μg/kg/min of remifentanil, and 20 mg/h of rocuronium. A 7 mm endotracheal tube was used for intubation, and 0.05 mg of phenylephrine or 4 mg of ephedrine were administered as needed for hypotension. Intraoperative monitoring included invasive arterial pressure, central venous pressure, patient state index (PSI), regional cerebral oxygen saturation, and transesophageal echocardiography. Circulatory dynamics were monitored using a FloTrac Sensor (Edwards Lifesciences Japan Ltd., Tokyo, Japan), which allowed for continuous monitoring of cardiac output. This device uses arterial pressure waveform analysis to calculate stroke volume and cardiac output. From anesthesia induction, cardiac output and the cardiac index ranged between 1.6–2.1 L/min and approximately 1.5–2.0 L/min/m^2^, respectively, while stroke volume variation remained around 12%–16% (Figure 4). The percutaneous MVC procedure was performed without any significant hemodynamic instability. Subsequently, percutaneous LAAC was performed. Throughout the procedure, 0.03 μg/kg/min of norepinephrine was administered continuously. Administration of norepinephrine was discontinued at the end of anesthesia administration after completion of the surgery. During the procedure, respiratory management was conducted with positive end‐expiratory pressure set at 5–10 cm H_2_O. Patient sedation was monitored using the PSI, which ranged from 20 to 40 throughout the procedure. Anesthesia duration was 2 h and 38 min, with a surgery duration of 1 h and 36 min. The total fluid input was 910 mL, urine output was 250 mL, and there was minimal blood loss. After surgery, 0.3 mg of flumazenil was administered, leading to awakening within 2 min. The patient was responsive, prompting extubation. After extubation, both respiratory and circulatory conditions remained stable, and the patient was transferred to the cardiac care unit. The patient's recovery was uneventful, and she was discharged on the 10th postoperative day (Figure 5A,B).

## 4 CONCLUSION AND RESULTS

Remimazolam appears to be a suitable agent for anesthesia management in elderly patients with impaired cardiac function because it can stabilize hemodynamics effectively. Furthermore, it can be used safely without causing events such as delayed awakening. However, this is a single case report, and we look forward to future clinical studies. Anesthesia management with remimazolam appeared to be beneficial in elderly patients with many comorbidities.

## 5 DISCUSSION

Surgery is generally indicated for severe MR. However, it has been reported that approximately 80% of patients with secondary MR do not undergo surgery due to factors such as advanced age, significant renal dysfunction, and increased perioperative risk from previous thoracotomy adhesions.^3^ Particularly in patients aged 80 years and older undergoing mitral valve surgery, a mortality rate of 36% has been reported.^4^ In recent years, transcatheter mitral valve repair has emerged as an alternative treatment for such cases, replacing traditional open‐heart surgery.^5^ Transcatheter mitral valve repair is less invasive than open‐heart surgery and is applicable even for elderly patients who are not candidates for open‐heart surgery. However, elderly patients often have various medical histories, and their physiological and organ functions may be diminished compared with healthy individuals. Therefore, it is essential to pay attention to hemodynamic stability during anesthesia induction. Remimazolam, an intravenous anesthetic that became available in Japan in 2020, causes less circulatory depression than propofol, another intravenous anesthetic.^6^ Thus, it is considered a suitable anesthetic for patients with anticipated hemodynamic instability, such as those with impaired cardiac function. In a study of different anesthetic agents in transcatheter treatment, Fechner J et al. reported that remimazolam reduced the amount of vasopressor agents more than propofol.^7^ In this case, FloTrac Sensor was used intraoperatively as an indicator of circulatory status, and anesthesia management was performed without complications. The use of FloTrac Sensor has been reported to improve clinical outcomes for patients undergoing major surgery.^8^ Furthermore, delirium is one of the postoperative complications in elderly patients.^9^ The onset of delirium increases the risk of postoperative cognitive dysfunction and dementia, significantly impacting prognosis. Benzodiazepines are generally considered a risk factor for postoperative delirium. Therefore, it is recommended that the use of benzodiazepines be minimized both before and during surgery.^10^, ^11^ However, to date, there is no evidence to suggest that remimazolam has a higher incidence of delirium than other sedatives.^12^, ^13^, ^14^ Furthermore, remimazolam has the unique characteristic of having an antagonist available, which allows for rapid reversal and awakening. Rapid awakening enables prompt assessment of paralysis or numbness, facilitating early detection of neurological complications such as cerebral hemorrhage or stroke. However, there have been reported cases where administration of flumazenil induced re‐sedation after awakening, highlighting the need for adequate postoperative observation, especially in elderly patients, or during prolonged surgeries. To prevent re‐sedation or delayed awakening, it is crucial to maintain appropriate dosing.^15^, ^16^ During surgery, it is desirable to adjust and manage the dosage of remimazolam as needed using brain function monitors such as BIS or Sedline.^17^ In addition, after surgery, it may be advisable to monitor oxygen saturation and other parameters for a certain period of time. In the present case, the patient awakened within approximately 1–2 min after administration of flumazenil and was responsive enough for extubation. She was able to converse without re‐sedation occurring. Combination of remimazolam and flumazenil accelerates recovery from general anesthesia and lowers the risk of respiratory depression compared to propofol.^18^ Remimazolam offers several advantages over traditional sedatives due to its short half‐life and the availability of an antagonist. It is likely to be increasingly used in various clinical settings in the future.

## AUTHOR CONTRIBUTIONS

**Sumika Yamaguchi:** Conceptualization; writing – original draft. **Yusuke Ishida:** Conceptualization; writing – original draft; writing – review and editing. **Tomomi Sasaki:** Writing – review and editing. **Satoshi Higuchi:** Writing – review and editing. **Kiyoko Bito:** Writing – review and editing. **Katsunori Oe:** Writing – review and editing.

## FUNDING INFORMATION

None.

## CONFLICT OF INTEREST STATEMENT

The authors declare no conflict of interest.

## CONSENT

Written informed consent was obtained from the patient to publish this report in accordance with the journal's patient consent policy.

## Data Availability

The data that support the findings of this study are available from the corresponding author upon reasonable request.
